# New Techniques for Preparing Thin Films: Characteristics and Further Developments

**DOI:** 10.3390/ma19214557

**Published:** 2026-10-29

**Authors:** Baiquan Liu, Peng Xiao

**Affiliations:** 1 Guangdong Provincial Key Laboratory of Display Materials and Technologies, State Key Laboratory of Optoelectronic Materials and Technologies, School of Electronics and Information Technology, Sun Yat-sen University, Guangzhou 510275, China; 2 Guangdong-HongKong-Macao Joint Laboratory for Intelligent Micro-Nano Optoelectronic Technology, School of Physics and Optoelectronic Engineering, Foshan University, Foshan 528225, China

## 1. Introduction and Special Issue Scope

This Special Issue, entitled “New Techniques for Preparing Thin Films: Characteristics and Further Developments”, addresses the development of preparation and modification strategies for functional thin films. Thin-film technologies enable optical, electrical, mechanical, and thermal behaviour to be tailored through the control of composition, microstructure, thickness, and layer architecture. This versatility supports applications in electronic and optoelectronic systems, energy conversion, environmental technologies, and surface protection. Achieving the desired performance requires an understanding of how preparation conditions determine the structural and chemical features responsible for material function. Compositional modification can alter film morphology and functional response, while the conditions favouring one performance criterion may differ from those favouring another (Contribution 1, 2). Dopant incorporation and thermal treatment further influence crystalline characteristics, phase constitution, and electrical behaviour, highlighting the need to consider chemical and structural control together (Contribution 3, 4). These relationships provide the scientific motivation for connecting advances in thin-film preparation with systematic characterisation and application-specific evaluation.

The papers collected in this Special Issue explore this connection through complementary experimental investigations and reviews. The scope encompasses deposition techniques, doping and compositional engineering, multilayer design, post-deposition treatments, and structural and functional characterisation. These topics are organised around a common objective: to understand how preparation and modification can be coordinated to obtain film characteristics suited to their intended functions. Particular attention is given to the relationships among processing conditions, composition, microstructure, and physical properties, which provide a basis for selecting suitable fabrication routes and identifying their limitations (Contribution 5, 6). The purpose of the collection is therefore to consolidate understanding across different material systems and connect material optimisation with practical requirements. At the device level, this perspective extends to interfacial integrity, environmental stability, mechanical compatibility, and the integration of complementary functions (Contribution 7, 8). Bringing these considerations together establishes a coherent framework for evaluating both functional performance and operational reliability. The Special Issue thus links fundamental thin-film science with engineering development and identifies directions for further progress in reproducible preparation, durable devices, and scalable fabrication.

## 2. An Overview of the Special Issue

The opening research article by Bancheva-Koleva and co-workers examines the effects of cerium incorporation on ZnO thin films prepared by thermal vacuum evaporation and subsequent annealing (Contribution 1). Structural, morphological, and optical characterisation showed that the films retained the hexagonal wurtzite structure, while doping modified their crystallinity, surface topography, and absorption behaviour. The nominally 2% Ce-doped film exhibited a compact morphology and a root-mean-square roughness of 7.52 nm, compared with 39.02 nm for undoped ZnO. Increasing the nominal Ce content to 5% produced grain agglomeration and increased roughness. Band-gap narrowing at 2% was followed by a slight increase at 5%. The 2% film also showed the highest methyl-orange degradation rate, which the authors associated with defect-mediated charge separation. These findings demonstrate the importance of controlling dopant concentration when balancing structural quality, optical absorption, and photocatalytic performance.

In the second contribution, Cholakova and co-workers investigate TiCN/ZrCN nanomultilayer coatings deposited by cathodic arc evaporation with a modulation period of approximately 12 nm (Contribution 2). Varying the N_2_/CH_4_ flow ratio enabled control of carbon incorporation and its effects on microstructure and tribo-mechanical behaviour. X-ray diffraction and Raman measurements indicated crystalline (Ti,Zr)(C,N) phases accompanied by amorphous carbon in the two coatings with carbon contents of 14.6 and 15.9 at.%, respectively. The coating containing 14.6 at.% carbon achieved the highest average hardness of 40 GPa, whereas the 15.9 at.% coating exhibited the lowest wear rate of 3.16 × 10^−6^ mm^3^/(N·m) under the selected dry-sliding conditions. Carbon incorporation improved hardness and wear resistance relative to the TiN/ZrN reference. The different compositions associated with maximum hardness and minimum wear highlight the need to balance mechanical and tribological responses when designing multilayer protective coatings.

The third article, by Ma and co-workers, reports the preparation of tungsten-doped hexagonal Ta_2_O_5_ films on sapphire substrates using metal–organic chemical vapour deposition (Contribution 3). Structural characterisation identified polycrystalline δ-Ta_2_O_5_ films whose preferred orientation depended on the W concentration. At 1.5% W, the films exhibited the best crystalline quality and a predominant (0001) orientation, whereas higher doping disrupted the preferential alignment. Hall measurements established n-type conduction in all doped samples. The 1.5% W-doped film displayed a minimum resistivity of 2.68 × 10^4^ Ω·cm, a carrier concentration of approximately 7.4 × 10^14^ cm^−3^, and an optical band gap of 3.92 eV. The study establishes W doping as a route to modifying the electronic behaviour of Ta_2_O_5_, while identifying improved crystal quality, carrier transport, and doping activation as priorities for its development as a semiconductor active-layer material.

In the fourth paper, Ren and co-workers examine annealing-induced phase evolution in Mn_1_._6_Zn_0_._2_Ni_0_._6_Mg_0_._2_Al_0_._4_O_4_ high-entropy oxide films prepared by electron-beam deposition of metallic multilayers followed by oxidation in air (Contribution 4). Annealing between 650 and 950 °C produced spinel-containing films, with a single spinel phase detected at 850 °C and secondary phases observed at the other temperatures. All samples exhibited negative-temperature-coefficient resistance behaviour consistent with small-polaron hopping. Importantly, the specimen annealed at 750 °C showed the highest temperature-sensitivity parameter, B_320_/_420_ = 3250.8 K, despite containing a secondary phase. This result should be distinguished from maximum electrical conductivity, since the same specimen also exhibited the highest measured resistance. The study therefore illustrates that phase purity and thermistor response do not necessarily reach their optimum under identical processing conditions, providing insight into thermal-treatment strategies for multicomponent oxide films.

The fifth contribution, a review by Geng and co-workers, examines the preparation of Bi_2_Te_3_-based thermoelectric thin films by magnetron sputtering (Contribution 5). The authors organise the discussion around deposition parameters, post-annealing treatments, and doping strategies, relating these variables to microstructure, stoichiometry, crystallographic texture, and thermoelectric transport. Particular attention is given to the competing effects of improved crystallinity, defect modification, and tellurium volatilisation during growth and thermal processing. The review also discusses how composition and interface engineering influence the balance among electrical conductivity, the Seebeck coefficient, and thermal conductivity. Rather than identifying a universally optimal processing condition, it emphasises the interdependence of these factors and the limited comparability of results obtained under different experimental conditions. The resulting synthesis provides guidance for process optimisation and identifies large-area uniformity, substrate compatibility, and operational stability as priorities for practical thermoelectric applications.

In the sixth article, Polis and co-workers review selected deposition techniques and doping strategies for ZnO thin films, with an emphasis on their optical and electrical properties (Contribution 6). The discussion compares spray pyrolysis, chemical bath deposition, spin coating, magnetron sputtering, chemical vapour deposition, and atomic layer deposition in terms of processing conditions, structural control, and application requirements. Representative doping studies illustrate how the synthesis route influences dopant incorporation, crystallinity, optical transparency, and carrier transport. The review highlights the need to balance conductivity and transparency against processing cost, thermal budget, and substrate compatibility when selecting a fabrication strategy. It also identifies persistent challenges involving stable p-type conductivity, environmental degradation, and interface control. By connecting deposition methods with compositional modification, the article provides a comparative framework for developing ZnO-based transparent conductors and related electronic and optoelectronic components.

The seventh contribution, by Li and co-workers, reviews thin-film encapsulation for OLEDs from the perspective of engineering validation and manufacturing (Contribution 7). The authors organise the assessment around basic properties, reliability, and compatibility, extending beyond water-vapour barrier performance to include stress, transparency, adhesion, particle coverage, and interactions with OLEDs and thin-film transistors. The discussion relates encapsulation failure to material degradation, mechanical damage, particle-induced defects, and lateral moisture ingress. Emerging strategies include atomic-layer-deposited barriers, inorganic/organic nanolaminates, stress management, multifunctional structures, and improved deposition equipment. Although these approaches offer opportunities for thinner and more flexible encapsulation, their implementation remains constrained by deposition throughput, maintenance requirements, and incomplete reliability validation. The review demonstrates why low permeability alone is insufficient to establish practical suitability and provides a framework for evaluating encapsulation technologies against the combined demands of flexible OLED products.

Finally, Marciel and co-workers review advances in electrochromic technology for multifunctional flexible devices (Contribution 8). The article connects material selection, deposition methods, and device architectures with optical modulation and complementary functions, including energy harvesting, energy storage, sensing, and thermal regulation. Conventional inorganic and organic electrochromic materials are considered alongside composites, hybrid systems, resonant structures, and emerging two-dimensional materials. Representative devices illustrate how reversible electrochemical processes can support colour switching while providing additional functionality, such as visual charge-state indication. The review also examines the challenges of maintaining ionic and electronic transport, interfacial integrity, and cycling stability during mechanical deformation. Scalable fabrication, suitable flexible transparent electrodes, and mechanically robust electrolytes remain important development needs. These considerations position multifunctional electrochromic devices as promising platforms for smart windows, wearable systems, and adaptive displays, while clarifying the requirements for broader practical implementation.

## 3. Conclusions

This Special Issue brings together eight contributions examining the relationships between thin-film preparation, structural and compositional modification, and application-specific performance. Covering photocatalytic oxides, protective coatings, semiconductor and thermistor films, thermoelectric materials, OLED encapsulation, and flexible electrochromic devices, the collection connects fundamental materials research with engineering requirements. Recent literature on ZnO deposition similarly emphasises the influence of preparation routes on film characteristics and functional behaviour [1]. Developments in deformable displays and flexible electrochromic energy storage further highlight the need to consider material properties together with device architecture and mechanical compatibility [2,3]. Collectively, these perspectives support evaluating thin-film advances through both the control of material characteristics and their contribution to reliable device operation.

A central insight is that effective optimisation requires coordinated control of processing, composition, and microstructure. The studies of Ce-doped ZnO and W-doped Ta_2_O_5_ demonstrate that functional responses depend on dopant concentration and associated structural changes (Contribution 1, 3). The TiCN/ZrCN and high-entropy oxide investigations further show that the conditions yielding maximum hardness or phase purity do not necessarily maximise wear resistance or the thermistor B-value (Contribution 2, 4). Related research on CrN and TiZrNbMo coatings reinforces the importance of deposition conditions in determining microstructure and mechanical properties [4,5]. In thermoelectric films, recent studies of vacancy modulation with Ti additives and W doping demonstrate additional routes for tailoring carrier transport and power factors [6,7]. These findings complement the Bi_2_Te_3_ and ZnO reviews (Contribution 5, 6) and favour application-specific optimisation over the pursuit of a single structural or compositional target.

Operational reliability remains a prerequisite for translating these improvements into practical technologies. The OLED encapsulation review emphasises that low initial water-vapour transmission alone cannot establish long-term protection (Contribution 7). Recent investigations show that hygrothermal exposure can impair both the mechanical integrity and barrier performance of atomic-layer-deposited Al_2_O_3_ [8]. Research on silicon nitride and oxynitride encapsulation also identifies moisture-induced changes in chemical bonding and stress as relevant indicators of stability [9]. Conversely, low-temperature Al_2_O_3_/TiO_2_ nanolaminates demonstrate how multilayer design can improve mechanical robustness and environmental resistance [10]. Together, these results support assessing chemical degradation, stress evolution, interfacial adhesion, and device lifetime within a common reliability framework. Clearly specified ageing conditions and mechanical test protocols are essential for meaningful comparison and for identifying failure mechanisms relevant to intended operating environments.

Beyond individual material improvements, the collection highlights the importance of integrating complementary functions without compromising stability. Flexible electrochromic systems require coordinated design of active films, electrodes, electrolytes, and interfaces (Contribution 8). Recent MXene-based microsupercapacitors demonstrate the integration of electrochromic response with energy storage in flexible devices [11], while nanostructured quasiplanar interfaces offer a route to improved switching behaviour and stability in inorganic electrochromic windows [12]. These examples reinforce the value of linking interfacial structure to both charge transport and device performance. However, successful laboratory demonstrations require further validation of uniformity, reproducibility, and durability before broader implementation. Collaboration among materials researchers, device engineers, and manufacturing specialists can help establish realistic specifications and evaluate performance at the level of complete devices.

Looking ahead, research should deepen the mechanistic understanding of dopant incorporation, phase evolution, and interfacial degradation while developing manufacturing-compatible preparation routes. Low-temperature sol–gel processing offers opportunities for integrating crystalline oxide films with thermally sensitive substrates [13], and inkjet printing provides a promising approach to patterned electrochromic devices [14]. In parallel, autonomous sputtering that combines optical emission spectroscopy with Bayesian optimisation demonstrates the potential of measurement-guided control over film composition [15]. Extending such approaches will require reproducible datasets, appropriate calibration, and experimental validation across processing conditions [16,17]. In situ characterisation and complementary functional measurements should accompany these developments to connect process signals with material behaviour. Finally, assessing durability alongside throughput, material consumption, energy requirements, and cost will help translate the scientific advances represented in this collection into practical thin-film technologies.
